# Design and Evaluation of a Compact CNN for EMG-Based Wearable Systems Under Embedded Constraints

**DOI:** 10.3390/s26123862

**Published:** 2026-06-17

**Authors:** Valentina Tirsu, Andrei Dorogan, Lilia Sava, Larisa Dunai, Alexandru Ilev, Nelea Manin

**Affiliations:** 1 Department of Telecommunications and Electronic Systems, Faculty of Electronics and Telecommunications, Technical University of Moldova, 2004 Chisinau, Moldova; valentina.tirsu@tse.utm.md (V.T.); andrei.dorogan@srco.utm.md (A.D.); lilia.sava@fet.utm.md (L.S.); nelea.manin@tse.utm.md (N.M.); 2 Department of Graphic Engineering, Universitat Politècnica de València, 46022 Valencia, Spain

**Keywords:** electromyography (EMG), wearable cyber-physical systems, TinyML, embedded ai, convolutional neural networks, signal classification, neuromuscular signal processing, resource-constrained devices

## Abstract

**Highlights:**

**What is the current knowledge on the topic?**

- EMG-based movement recognition is increasingly used in wearable and cyber–physical systems.
- Embedded deployment requires AI models with low memory footprint and computational complexity.

**What are the main findings?**

- A compact 1D-CNN (~2000 parameters, ~2 KB after quantization) achieved approximately 91% classification accuracy under embedded constraints.
- The proposed CPS-oriented framework integrates EMG acquisition, preprocessing, and real-time AI inference while maintaining suitability for TinyML deployment.

**Abstract:**

Electromyographic (EMG) signals are increasingly used in wearable cyber–physical systems (CPS), where reliable movement recognition must be achieved under limited computational resources. In this study, we present a compact EMG processing framework that integrates signal acquisition, preprocessing, segmentation, and movement classification within a unified pipeline designed for embedded-oriented applications. The proposed approach combines a multi-channel EMG acquisition system with a lightweight one-dimensional convolutional neural network (1D CNN) developed according to TinyML principles, withprocessing input windows of size 32 × 3 and low computational complexity and memory requirements. Experimental evaluation was conducted on a dataset collected from 15 participants performing squat, walking, and running activities under realistic acquisition conditions. The proposed model achieved an accuracy of 0.9135, an F1-score of 0.9124, and a ROC AUC of approximately 0.96, demonstrating reliable classification performance. Following 8-bit quantization, the model size was reduced to approximately 2 KB, supporting deployment on resource-constrained embedded platforms. The results show that compact CNN architectures can effectively classify EMG-based movement patterns while maintaining a small computational footprint, providing a practical foundation for future wearable CPS and TinyML-enabled applications.

## 1. Introduction

Electromyographic (EMG) signals provide valuable information about muscle activity and are widely used to infer human movement intention in rehabilitation, assistive technologies, prosthetic control, and human–machine interaction systems. Recent advances in wearable technologies and cyber–physical systems (CPS) have further expanded the role of EMG sensing in real-time biomedical applications, where reliable interpretation of neuromuscular activity is essential.

The growing adoption of machine learning techniques has significantly improved the analysis of EMG signals. In particular, convolutional neural networks (CNNs) have demonstrated strong performance by automatically learning discriminative patterns from multi-channel time-series data. Unlike traditional approaches based on manually engineered features, CNN-based models can extract relevant representations directly from raw signals. At the same time, the emergence of TinyML has created new opportunities for deploying compact artificial intelligence models on low-power embedded devices, enabling real-time processing under limited memory and computational resources [1].

Despite these advances, robust EMG classification remains challenging in real-world environments. Signal characteristics can be affected by factors such as electrode placement, muscle fatigue, acquisition noise, and physiological differences between users. Subject-independent classification is particularly difficult because muscle activation patterns and anatomical characteristics vary considerably across individuals [2,3,4]. As a result, models that perform well on known users often experience reduced performance when evaluated on previously unseen subjects.

Another challenge concerns the increasing complexity of many deep learning solutions. Although large neural network architectures can achieve excellent classification accuracy, they often require substantial computational resources and memory capacity. Such requirements may limit their applicability in wearable and embedded systems, where computational efficiency, memory footprint, and energy consumption are critical design constraints. Consequently, achieving a balance between classification performance and implementation efficiency remains an important research objective [5].

The limited availability of diverse EMG datasets further complicates this problem. Variations in age, gender, physiological characteristics, and acquisition conditions can significantly influence muscle activation patterns and affect model generalization. Therefore, evaluating compact AI models under realistic acquisition conditions is an important step toward the development of practical wearable systems.

In this study to address these challenges, we propose a compact EMG processing framework that integrates multi-channel signal acquisition, preprocessing, segmentation, and movement classification within a unified workflow. The proposed approach employs a lightweight one-dimensional convolutional neural network (1D CNN) containing approximately 2000 trainable parameters, following a TinyML-oriented design strategy aimed at reducing memory and computational requirements.

The framework was evaluated using EMG recordings collected from 15 participants performing squat, walking, and running activities under realistic acquisition conditions. The obtained results indicate that reliable movement classification can be achieved using a compact neural network architecture while maintaining a small memory footprint suitable for future embedded deployment.

The objective of this work is to investigate the feasibility of lightweight machine learning models for EMG-based movement recognition in resource-constrained environments. Rather than introducing a novel deep learning architecture, the study focuses on the design, implementation, optimization, and evaluation of a compact solution intended for future wearable applications.

The main contributions of this study are as follows:

(i) The development of a compact 1D CNN architecture for multi-channel EMG classification under embedded constraints;

(ii) The design of an integrated EMG processing pipeline combining signal acquisition, preprocessing, segmentation, and AI-based inference;

(iii) The implementation of a TinyML-oriented optimization workflow, including model quantization and TensorFlow Lite conversion;

(iv) The evaluation of the proposed framework from both classification and deployment perspectives within a wearable CPS context.

The remainder of this paper presents the proposed framework, experimental methodology, classification results, embedded implementation analysis, and a discussion of its applicability to future wearable cyber–physical systems.

## 2. Architecture of the Proposed Cyber–Physical System

The proposed cyber–physical system (CPS) combines electromyographic (EMG) signal acquisition, signal processing, artificial intelligence (AI), and embedded computing within a unified framework. Its purpose is to transform neuromuscular activity into meaningful information that can be used to recognize movement patterns and support intelligent decision-making in wearable applications.

Unlike conventional EMG analysis approaches that rely primarily on offline processing, the proposed architecture incorporates AI-based inference directly into the processing chain. This enables near real-time interpretation of EMG signals and allows movement recognition results to be used as part of a decision-support mechanism for wearable assistive systems [6,7]. Such an approach is particularly important in applications requiring low latency and efficient operation on resource-constrained hardware.

The architecture consists of five functional layers. The first layer performs EMG sensing and signal acquisition through surface electrodes positioned on lower-limb muscles. The second layer is responsible for preprocessing and segmentation, where signals are validated, normalized, and transformed into fixed-length temporal windows suitable for machine-learning analysis. The third layer performs AI-based analysis using a compact one-dimensional convolutional neural network (1D CNN) with approximately 2000 trainable parameters. The model processes input tensors of size 32 × 3 and was specifically designed to balance classification performance with computational efficiency. The fourth layer represents the decision logic, where classification outputs are interpreted and assigned to predefined movement categories. In the present study, three movement classes were considered: squat, walking, and running. The fifth layer supports embedded-oriented implementation through model quantization and preparation for deployment on resource-constrained platforms.

**Figure 1:**
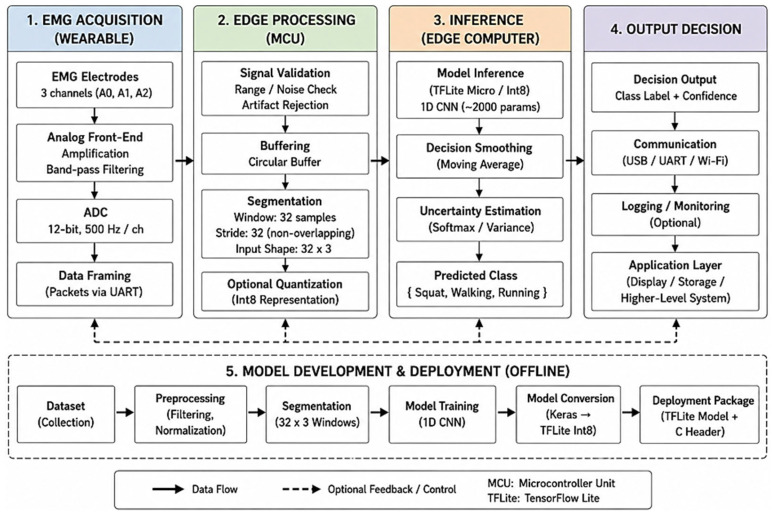
Conceptual architecture of the EMG-based wearable cyber–physical system.

The overall workflow of the proposed system is illustrated in Figure 1. The architecture integrates signal acquisition, preprocessing, AI-based classification, and decision-making within a single CPS framework, providing a practical foundation for efficient EMG-based movement recognition in wearable environments and future TinyML-enabled applications.

A host development layer supports data acquisition, debugging, and inference execution through a UART interface using TensorFlow 2.15.0 and Keras 2.15.0 (Google LLC, Mountain View, CA, USA). In the current implementation, EMG signals are acquired using a MyoWare 2 Muscle Sensor Development Kit (KIT-27920; SparkFun Electronics, Niwot, CO, USA) connected to an Arduino MEGA (WIZI Electronics Co., Ltd., Shenzhen, China), while movement classification is performed on a BPI-M2-BERRY v1.0 device (Shenzhen SinoVoIP Co., Ltd., Shenzhen, China). This hybrid configuration provides a practical environment for evaluating the complete acquisition and processing pipeline under realistic operating conditions while remaining relevant to future embedded implementations.

Information flows bidirectionally through the system. EMG signals generated by muscular activity are acquired, processed, and classified, while the resulting outputs can be used to support adaptive responses in wearable assistive applications. To improve stability, the architecture incorporates temporal smoothing and uncertainty-aware processing, reducing short-term fluctuations between predicted movement classes.

To facilitate deployment on resource-constrained platforms, the CNN model is quantized using an 8-bit integer representation, reducing its size to approximately 2 KB while preserving classification performance. The architecture also includes a reliability layer responsible for signal validation, protected model storage, and fail-safe mechanisms intended to maintain stable operation under abnormal conditions.

Security, resilience, and fail-safe mechanisms are included in the conceptual architecture as design considerations for future embedded implementations. These components were not experimentally implemented or evaluated in the present study and therefore should be regarded as prospective extensions rather than validated contributions. Consequently, the primary focus of this work remains the development and evaluation of a compact EMG classification framework.

Overall, the proposed architecture demonstrates how compact AI models can be integrated into wearable cyber–physical systems to enable efficient EMG signal interpretation with low computational and memory requirements. The resulting framework provides a practical foundation for future wearable applications requiring real-time movement recognition and energy-efficient operation.

**Figure 2:**
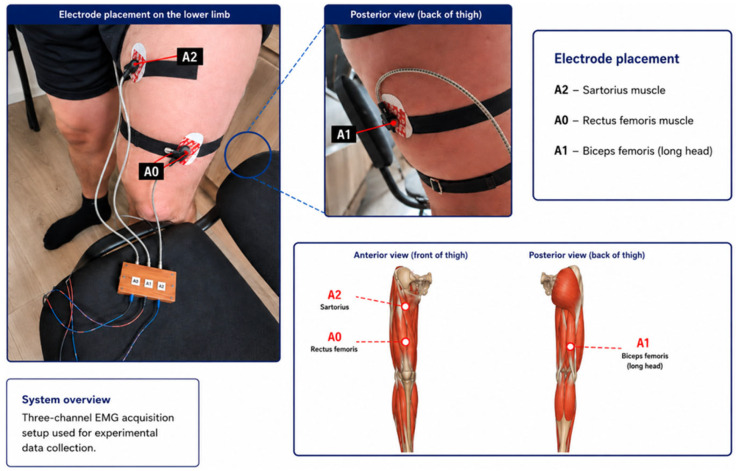
Experimental three-channel EMG acquisition setup and anatomical locations of the monitored muscles: Sartorius (A2), Rectus femoris (A0), and Biceps femoris (long head) (A1).

Electromyographic (EMG) signals were acquired using a three-channel configuration (A0–A2), with electrodes placed on the Sartorius, Rectus femoris, and Biceps femoris (long head) muscles. The electrode placement configuration used in the experimental setup is illustrated in Figure 2.

Surface EMG electrodes were positioned according to standard placement guidelines (e.g., SENIAM), aligned with the muscle fiber direction and separated by approximately 20 mm to ensure reliable signal acquisition [8,9]. Signals were recorded at a sampling frequency of 500 Hz, providing sufficient temporal resolution for capturing muscle activation dynamics while remaining compatible with embedded-oriented processing requirements. The acquired EMG signals were subsequently validated and preprocessed before machine-learning analysis.

Data Validation and Preprocessing. Prior to model development, the raw EMG recordings underwent a validation and preprocessing stage aimed at ensuring signal reliability. Incomplete or corrupted recordings were removed, and synchronization across the three analog channels (A0–A2) was verified to ensure correct multi-channel acquisition.

To reduce the influence of noise, artifacts, and outliers, a rule-based filtering procedure was applied using amplitude thresholds in the range of 800–5000 ADC units combined with signal quality criteria. These thresholds were selected empirically following visual inspection of multiple recordings and preliminary experiments designed to identify corrupted, saturated, or low-quality signals while preserving valid muscle activity. Values below 800 ADC units were typically associated with weak or noisy recordings, whereas values above 5000 ADC units frequently indicated signal saturation or acquisition artifacts. This procedure enabled the exclusion of recordings affected by electromagnetic interference, electrode displacement, or insufficient signal quality.

After validation, the signals were segmented and organized into a multi-channel representation suitable for machine learning processing. The input data were represented as $X\;\in \;R^{32\times 3}$, where 32 corresponds to the number of temporal samples and 3 to the number of EMG channels. This representation preserves both temporal dynamics and spatial information related to muscular activity while remaining well suited for one-dimensional convolutional neural network (1D CNN) processing [10,11].

The filtering procedure revealed the variability typically encountered during real-world EMG acquisition using the MyoWare 2 Muscle Sensor Development Kit (KIT-27920; SparkFun Electronics, Niwot, CO, USA). Recordings were excluded when channel degradation, low signal amplitude, signal saturation, or partial channel loss prevented reliable analysis. Although this resulted in relatively high rejection rates, the adopted strategy improved dataset quality and reduced the influence of corrupted signals on subsequent model training and evaluation.

Overall, the validation and preprocessing stage improved data consistency and provided a reliable foundation for subsequent EMG signal analysis and classification under realistic operating conditions [8].

AI-Based Analysis Methods. The artificial intelligence (AI) layer is responsible for identifying movement-related patterns from preprocessed multi-channel EMG signals by exploiting both their temporal and spatial characteristics. This enables reliable differentiation between movement phases and provides the information required for movement recognition within the proposed wearable system.

A one-dimensional convolutional neural network (1D CNN) was selected because of its ability to efficiently process multi-channel time-series data. Unlike conventional approaches based on handcrafted features, the CNN automatically learns relevant signal representations directly from EMG segments, reducing the need for manual feature engineering and improving robustness to noise and signal variability.

The proposed model follows a compact TinyML-oriented design with approximately 2000 trainable parameters. The architecture was developed to balance classification performance with computational efficiency while maintaining low memory and processing requirements. The model was implemented using TensorFlow and Keras and subsequently optimized through post-training quantization. The quantized model was then converted into a C-compatible representation using tinymlgen 0.2 (Eloquent Arduino, Italy) to facilitate execution on resource-constrained platforms.

Overall, the AI layer provides an efficient solution for EMG signal classification, enabling near-real-time operation while preserving the low-complexity characteristics required for wearable embedded systems.

Adaptive Control Methods. The classification output generated by the CNN is integrated into a decision layer that interprets detected movement patterns and enables adaptive responses. In this way, model inference contributes not only to signal analysis but also to the interaction between signal acquisition, processing, and system response within the cyber–physical system.

**Figure 3:**
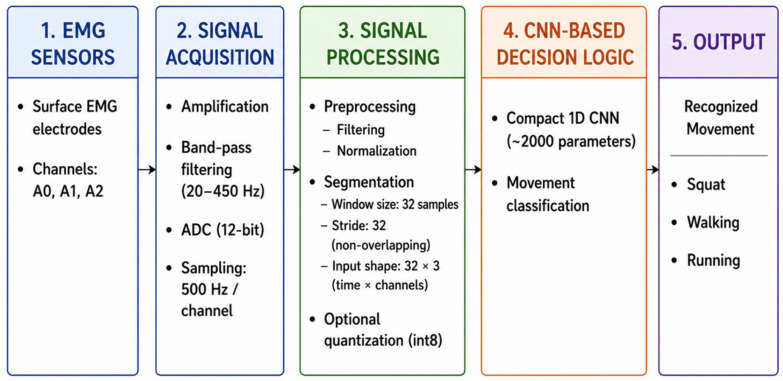
Experimental EMG processing pipeline used for movement classification.

The integration of the CNN model within the feedback-oriented CPS architecture is illustrated in Figure 3.

Multi-channel EMG signals are processed to identify relevant patterns of muscle activity, while the resulting classification outputs can support adaptive actions in wearable applications. In the current implementation, inference was performed on a BPI-M2-BERRY v1.0 platform, enabling execution of the complete processing pipeline under realistic operating conditions. This configuration demonstrates the feasibility of EMG-based movement recognition and provides a foundation for future deployment on embedded wearable platforms.

**Figure 4:**
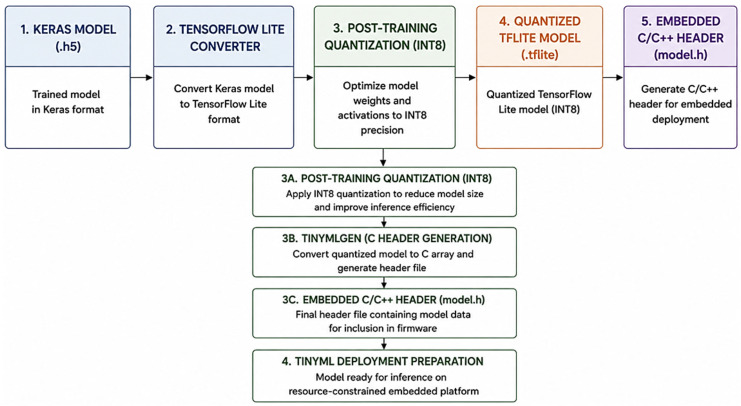
Conversion workflow of the CNN 1D model for TinyML deployment.

Embedded Implementation Methods. To enable execution in resource-constrained environments, the CNN model developed in TensorFlow and Keras was optimized through post-training quantization and adapted for embedded deployment. The conversion and optimization workflow is illustrated in Figure 4, which summarizes the steps required to transform the trained model into a format suitable for embedded applications.

The first step consisted of applying post-training 8-bit quantization (int8) to the trained model. This process reduced the model size from approximately 7.8 KB to about 2 KB while preserving classification performance.

The quantized model was subsequently converted into a C-compatible representation using the tinymlgen library, allowing integration into embedded firmware. Similar workflows are commonly used in TinyML applications targeting microcontroller-based systems [5].

To better understand the hardware requirements of the proposed model, the Arduino MEGA platform was considered as an example of an ultra-low-resource device. As summarized in Table 1, its limited SRAM capacity and computational resources impose significant constraints on direct neural network inference.

In the experimental configuration, Arduino MEGA was used for EMG signal acquisition and data transmission, while inference was performed on a BPI-M2-BERRY v1.0 platform. This hybrid setup enabled evaluation of the complete acquisition, preprocessing, and classification pipeline under realistic operating conditions while allowing the computationally intensive inference stage to be executed on more capable hardware.

Considering the final model size (~2 KB) and the estimated computational complexity (~3 × 10^4^ operations per inference), the proposed architecture appears suitable for deployment on resource-constrained embedded platforms. This assessment is based on the memory footprint and computational requirements of the quantized model. However, experimental validation on microcontroller-based hardware remains part of future work.

**Figure 5:**
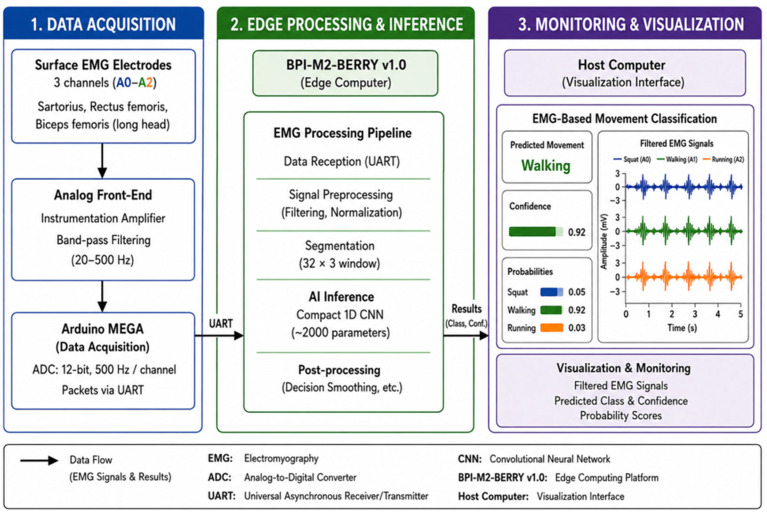
Experimental setup for EMG data acquisition, inference on the BPI-M2-BERRY v1.0 platform, and result monitoring.

The experimental setup used during system evaluation is illustrated in Figure 5.

The results demonstrate that compact CNN models can provide reliable EMG classification while meeting the resource constraints of embedded platforms, supportings their potential use in future wearable and TinyML-enabled applications.

## 3. Experimental Setup and Evaluation Methodology

The experimental dataset was collected from 15 healthy male participants aged between 18 and 21 years. A relatively homogeneous participant group was intentionally selected during the initial stage of the study to reduce physiological variability and facilitate evaluation of the proposed EMG classification framework. All participants performed the acquisition protocol under similar experimental conditions.

Data collection sessions lasted approximately 5–10 min per participant and included 60–80 repetitions of lower-limb movements separated by short pauses of 1–5 s. The protocol was designed to capture representative muscle activation patterns while maintaining consistency across recordings.

Three movement classes were considered: squat, walking, and running. Additional transition movements, such as kneeling and standing, were recorded but were not treated as independent classes during model training and evaluation.

The initial dataset consisted of 1011 multi-channel EMG recordings acquired from three monitored muscles. Each recording contained approximately 64–66 samples per channel and was stored as an individual acquisition buffer. Following validation and quality filtering, the recordings were segmented into consecutive non-overlapping windows of 32 samples, generating the EMG segments used for model training and evaluation.

The collected dataset reflects realistic EMG acquisition conditions and includes variability associated with electrode placement, movement execution, muscle activation patterns, and inter-subject differences. Consequently, it provides a suitable basis for evaluating classification performance and the feasibility of future wearable implementations.

Movement Protocol: To ensure consistency across recordings and reduce ambiguity between movement phases, participants performed each activity at a controlled rhythm. Short pauses (1–5 s) were introduced between active and resting phases to facilitate signal segmentation and improve repeatability.

**Figure 6:**
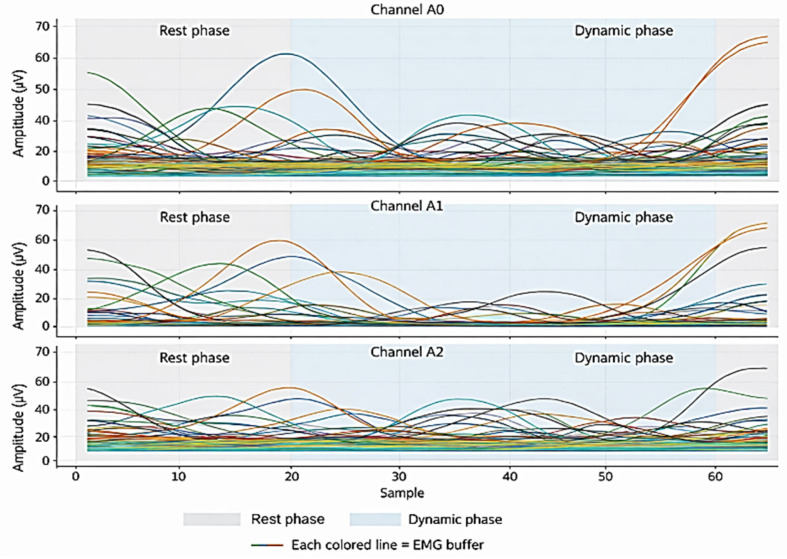
Representative EMG signal segments recorded on three channels (A0–A2) over a 64-sample temporal window.

Representative EMG signal segments recorded across the three acquisition channels are illustrated in Figure 6. The figure highlights the differences in signal amplitude and temporal behavior observed during the monitored activities.

Each representative recording shown in Figure 6 contains approximately 64 samples per channel. During preprocessing, these recordings were subsequently divided into fixed-length windows of 32 samples before being provided as input to the CNN model.

The observed signal patterns demonstrate clear variations in muscle activation across movement phases, providing discriminative information that can be exploited by machine learning algorithms for movement classification.

Data Preprocessing and Evaluation Protocol: To prepare the EMG data for model training and evaluation, a preprocessing stage was applied to ensure compatibility with the proposed 1D CNN architecture and improve numerical stability. The raw signals were verified for dimensional consistency, converted to float32 format, and normalized to reduce amplitude variations between recordings.

**Figure 7:**
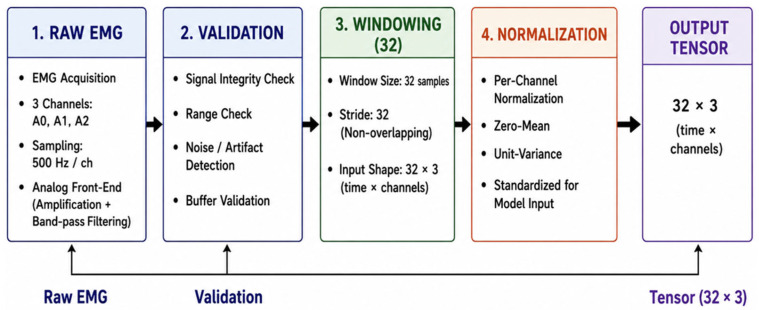
EMG data preprocessing workflow.

The preprocessing workflow applied to the EMG signals is illustrated in Figure 7.

A non-overlapping segmentation strategy (stride = 32) was adopted to reduce redundancy and computational complexity while preserving the temporal characteristics of the analyzed movements.

For model development, the dataset was initially divided into training (70%) and validation (30%) subsets using a stratified random split, preserving the class distribution. However, this approach may introduce subject overlap between the two subsets.

To assess subject-independent generalization, a leave-one-subject-out (LOSO) cross-validation protocol was applied. In each iteration, the model was trained on data from N−1 participants and tested on the remaining unseen participant. The results were reported as mean ± standard deviation, with model reinitialization performed at every iteration to prevent information leakage.

Additionally, stratified 5-fold cross-validation was used to provide a more robust estimate of model performance. While stratified cross-validation evaluates model consistency across different data partitions, LOSO offers a more realistic assessment of wearable EMG systems operating with previously unseen users.

## 4. Design and Training of the CNN Model

To classify EMG signals, a one-dimensional convolutional neural network (1D CNN) was adopted due to its ability to efficiently process multi-channel time-series data. Unlike traditional approaches based on handcrafted features, CNNs automatically learn relevant temporal patterns directly from the input signals, making them well suited for wearable sensing applications.

The network input consisted of windows of size 32 × 3, representing 32 consecutive samples acquired from three EMG channels. The architecture was designed according to TinyML principles to balance classification performance, memory footprint, and computational complexity. The resulting network structure is summarized in Table 2.

Several CNN configurations were investigated during preliminary experiments, and the final architecture was selected as a compromise between classification performance and deployment feasibility. Increasing the number of filters or network depth produced only marginal improvements in accuracy while increasing computational complexity and memory requirements. Consequently, a compact architecture containing approximately 2000 trainable parameters was considered the most suitable solution.

Model training was performed using the Adam optimizer with a learning rate of 0.001, a batch size of 32, and 300 epochs. Categorical cross-entropy was used as the loss function. The selected hyperparameters were determined empirically and provided stable convergence during training. Loss values decreased rapidly during the initial epochs and stabilized after approximately 150–200 epochs. Categorical cross-entropy was selected instead of mean squared error (MSE) because the task involves multi-class classification with softmax outputs. Cross-entropy directly measures the discrepancy between predicted class probabilities and ground-truth labels, providing faster convergence and more appropriate optimization behavior for classification problems than regression-oriented losses such as MSE.

The evaluation protocols, including stratified train–validation partitioning, stratified 5-fold cross-validation, and leave-one-subject-out (LOSO) validation, are described in Section 3.

To facilitate execution on resource-constrained platforms, post-training 8-bit quantization (int8) was applied, which reduced the model size from approximately 7.8 KB to 2.0 KB while preserving classification performance. The trained model was converted from TensorFlow/Keras to TensorFlow Lite and exported to a C-compatible representation using the tinymlgen library.

Although the converted model is compatible with resource-constrained microcontroller platforms, experimental validation was performed on a BPI-M2-BERRY v1.0 platform. Deployment on MCU-class hardware remains subject to future work.

**Figure 8:**
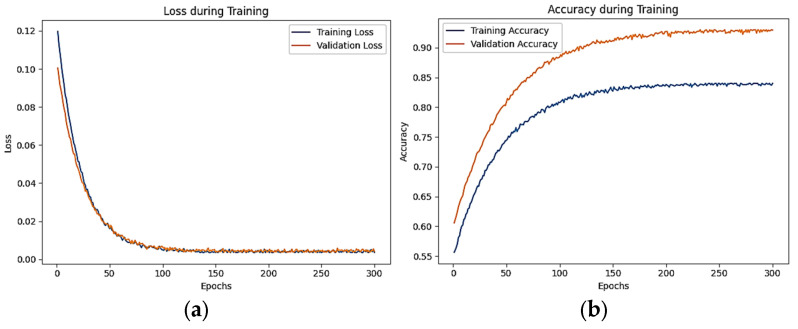
Training and validation performance of the CNN model: (**a**) loss evolution and (**b**) classification accuracy over training epochs.

The computational cost of the proposed network is approximately 3 × 10^4^ MAC operations per inference, which the total RAM requirement is estimated at approximately 3.5–4 KB. These values should be considered indicative rather than experimentally measured. Figure 8 presents the evolution of training and validation loss together with classification accuracy, illustrating stable convergence and the absence of significant overfitting.

## 5. Performance Evaluation and Comparative Analysis

Classification Metrics: The performance of the proposed CNN model was evaluated using standard classification metrics, including accuracy, precision, recall, F1-score, and the false positive rate (FPR). Together, these indicators provide a comprehensive assessment of the model’s ability to recognize movement-related patterns in EMG signals.

The overall results are summarized in Table 3. The model achieved an accuracy of 0.9135, with balanced precision (0.9097) and recall (0.9152), resulting in an F1-score of 0.9124. The low false positive rate (≈0.06) indicates that incorrect classifications were relatively infrequent, an important characteristic for wearable systems that rely on reliable movement recognition.

The average inference time measured on the desktop platform was approximately 0.6 ms per input window. Based on the computational complexity of the quantized model (≈3 × 10^4^ operations per inference), the expected latency on embedded hardware was estimated to range between 5 and 15 ms. These values represent analytical estimates derived from model characteristics and are intended to indicate deployment feasibility rather than experimentally measured embedded performance.

To further evaluate the effectiveness of the proposed approach, a comparative analysis was conducted using three widely adopted machine-learning methods: Support Vector Machine (SVM), Random Forest (RF), and k-Nearest Neighbors (k-NN). All baseline classifiers were trained using the same normalized EMG segments employed by the proposed CNN model and were evaluated using an identical stratified train–validation split. Hyperparameters were selected through preliminary tuning and kept fixed throughout the evaluation process. This comparison was used to assess whether the proposed CNN architecture provides advantages over conventional machine-learning approaches under equivalent evaluation conditions. Table 4 summarizes the results.

The proposed CNN achieved the highest overall performance among the evaluated methods. Although the differences in accuracy were relatively modest, the CNN consistently obtained superior recall and F1-score values, indicating an improved ability to capture the temporal relationships present in multi-channel EMG signals.

**Figure 9:**
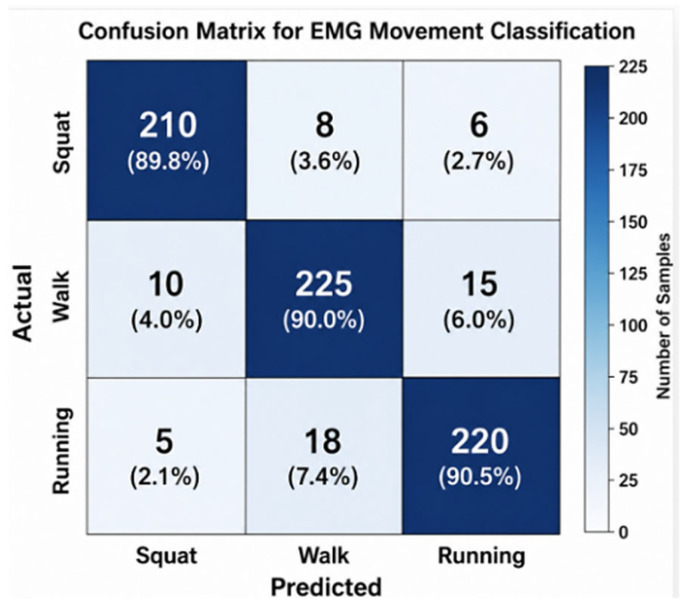
Confusion matrix for multi-class EMG movement classification (squat, walk, running).

The confusion matrix shown in Figure 9 illustrates the distribution of classification outcomes across the three movement classes. Most samples are concentrated along the main diagonal, indicating a high proportion of correctly classified observations across all categories.

To provide a more detailed evaluation, per-class performance metrics are presented in Table 5.

The per-class results indicate balanced performance across all movement categories. Slightly higher scores were obtained for the running class, whereas walking produced the lowest values among the three activities. Nevertheless, precision, recall, and F1-score remained relatively consistent across all classes, suggesting stable classification performance despite the unequal distribution of samples within the dataset.

Most classification errors occurred between walking and running activities. This behavior is expected because both movements involve cyclic lower-limb activation patterns and partially overlapping EMG characteristics. Although running generally produces stronger and more regular muscle activation, transitional gait phases may generate similar signal patterns, increasing the likelihood of misclassification. Similar findings have been reported in previous EMG-based movement recognition studies, where biomechanically related dynamic activities represent the most challenging classes to separate. 

The dataset exhibited a moderate imbalance among movement classes, with walking represented by a smaller number of validated segments after quality filtering. To mitigate the influence of class imbalance during model development, stratified partitioning was applied in both the train–validation split and the 5-fold cross-validation procedures, ensuring that class proportions were preserved across all evaluation subsets. Performance was additionally assessed using precision, recall, and F1-score metrics rather than accuracy alone, providing a more balanced evaluation under unequal class distributions. Although no explicit class-weighting or oversampling techniques were applied, the relatively consistent per-class F1-scores suggest that the model was not strongly biased toward the majority classes.

Experimental Results: The learning curves presented in Figure 8 demonstrate stable convergence throughout the training process. The loss decreased rapidly during the initial training epochs and gradually stabilized after approximately 150–200 epochs. Similarly, the training and validation accuracy curves followed comparable trajectories, indicating consistent learning behavior and the absence of significant overfitting.

The performance metrics presented in the previous section further confirm the ability of the proposed model to accurately distinguish between the considered movement classes while maintaining stable behavior across multiple evaluation measures.

From a computational perspective, the average inference time for an input window of size 32 × 3 was experimentally measured at approximately 0.6 ms on a desktop platform. Based on the computational complexity of the quantized model (≈3 × 10^4^ operations per inference), the expected latency on embedded hardware was estimated to range between 5 and 15 ms. Although these values were not experimentally measured on MCU-class devices, they indicate that the proposed model is suitable for near real-time operation in wearable applications.

**Figure 10:**
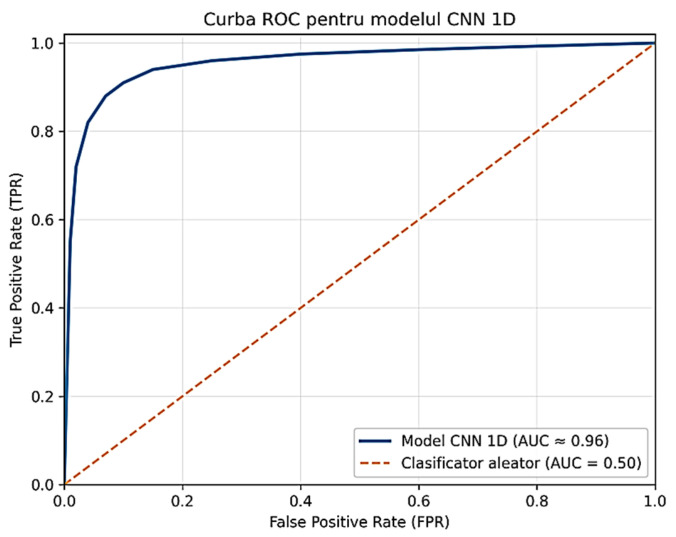
ROC curve (one-vs-rest) for multi-class EMG movement classification.

To further assess classification performance, a Receiver Operating Characteristic (ROC) analysis was performed using a one-vs-rest strategy for multi-class classification. The ROC AUC value was computed using a stratified train–validation evaluation protocol and therefore reflects segment-level classification performance. The resulting ROC curves are shown in Figure 10.

To evaluate the robustness of the proposed approach, a stratified 5-fold cross-validation procedure was performed while preserving the class distribution across all folds. In each iteration, four folds were used for training, with one fold for validation. As summarized in Table 6, the model achieved a mean accuracy of 0.936 ± 0.011, a precision of 0.920 ± 0.008, a recall of 0.902 ± 0.009, and an F1-score of 0.910 ± 0.007. The low standard deviations observed across folds indicate stable and consistent performance under different data partitions.

The confusion matrix presented in Figure 9 demonstrates strong classification capability, with most samples correctly assigned to their corresponding activity classes. Misclassifications were limited and occurred mainly between walking and running, which exhibit similar temporal and muscular activation characteristics. Such behaviour is expected due to the overlap in EMG patterns associated with these dynamic movements.

Overall, the obtained results confirm that the proposed compact CNN architecture provides accurate and computationally efficient classification of multi-channel EMG signals, making it suitable for resource-constrained wearable applications.

To further investigate subject-independent generalization, a leave-one-subject-out (LOSO) cross-validation protocol was conducted. In this evaluation, data from one participant were excluded from training and used exclusively for testing, while the model was trained on data from the remaining participants. The procedure was repeated for all 15 participants, with the model reinitialized and retrained at each iteration to ensure complete separation between training and testing data.

The LOSO results, summarized in Table 7, show a decrease in performance compared with segment-level validation, yielding an accuracy of 0.63 ± 0.04, precision of 0.59 ± 0.05, recall of 0.57 ± 0.04, and F1-score of 0.54 ± 0.05. This reduction is expected and reflects the considerable inter-subject variability inherent in EMG-based systems, including differences in muscle anatomy, electrode positioning, signal amplitude, and movement execution patterns.

Despite the lower performance, the LOSO evaluation provides a more stringent and realistic assessment of model behavior in practical deployment scenarios involving previously unseen users. The results indicate that, while the proposed framework achieves strong performance when representative training data are available, additional strategies such as subject adaptation, transfer learning, or larger and more diverse datasets may further improve cross-subject generalization.

The combination of stratified and LOSO validation protocols provides a comprehensive assessment of the proposed model, demonstrating both its classification effectiveness and its current limitations regarding subject-independent generalization in real-world wearable cyber-physical systems.

Limitations and Reliability Analysis: Although the experimental results demonstrate the consistent performance of the proposed 1D CNN model for EMG-based movement classification, several factors may influence its generalization capability and practical deployment.

The dataset was collected from 15 participants under controlled acquisition conditions. While stratified segment-level validation provided balanced class representation and stable performance estimates, it may allow data from the same participant to appear in both training and validation sets. To address this limitation, an additional LOSO evaluation was performed. The lower LOSO performance highlights the challenges associated with subject-independent EMG classification and confirms the need for larger and more diverse datasets.

The LOSO evaluation revealed a substantial reduction in performance (accuracy = 0.63 ± 0.04) compared with segment-level validation and stratified cross-validation, indicating that the proposed model currently exhibits limited subject-independent generalization and that performance is strongly influenced by inter-subject variability. Differences in muscle anatomy, physiological characteristics, electrode positioning, skin impedance, and movement execution patterns may significantly alter EMG signal distributions across users. Consequently, although the proposed framework demonstrates reliable performance when representative training data are available, its direct application to previously unseen users remains challenging. Future work will focus on improving cross-subject robustness through larger and more diverse datasets, domain adaptation techniques, transfer learning approaches, and subject-specific calibration procedures.

EMG signals are inherently affected by factors such as electrode placement, muscle fatigue, movement execution variability, and acquisition noise. These sources of variability can influence classification performance, particularly when the system is applied to previously unseen users. Nevertheless, the use of experimentally acquired EMG data allows the proposed framework to be evaluated under conditions relevant to wearable applications.

The analysis of prediction behavior showed stable classification during sustained movement phases, while increased variability was observed near movement transitions, where rapid changes in muscle activation occur.

Table 8 summarizes the main factors affecting model robustness and the mitigation strategies adopted in the proposed framework.

From a methodological perspective, the adopted evaluation strategy combines stratified validation, cross-validation, and subject-independent LOSO testing, providing a comprehensive assessment of model performance under different validation scenarios.

From a computational perspective, the compact architecture and quantized implementation support deployment on resource-constrained embedded platforms. The experimental workflow, which combines microcontroller-based signal acquisition with embedded-oriented inference, enables evaluation of the complete processing pipeline under realistic operating conditions.

Overall, the obtained results indicate that compact CNN architectures can effectively extract movement-related information from multi-channel EMG signals while maintaining low computational requirements. The proposed framework provides a practical basis for future wearable cyber–physical systems and highlights directions for improving subject-independent generalization.

Comparison with Related Work: Recent studies have demonstrated the effectiveness of machine learning and deep learning methods for EMG-based motion recognition. Conventional classifiers provide low computational complexity but often require manual feature engineering, whereas deep CNN architectures achieve higher classification performance at the cost of increased computational requirements [9,12,13,14,15].

More recently, research has focused on lightweight and TinyML-oriented solutions that enable EMG processing on resource-constrained embedded platforms [16,17,18]. These approaches reduce model size and inference cost while preserving practical classification performance, making them suitable for wearable applications.

To position the proposed framework within the current state of the art, Table 9 presents a qualitative comparison of representative EMG classification approaches.

Thise comparison indicates that high classification performance is often achieved at the cost of increased model complexity, whereas lightweight approaches emphasize computational efficiency and embedded deployment. The proposed framework follows the latter approach, combining a compact 1D CNN with approximately 2000 trainable parameters and an embedded-oriented processing workflow.

Rather than focusing solely on classification accuracy, the proposed solution balances performance and deployment requirements. The results demonstrate that reliable EMG classification can be achieved with a compact architecture suitable for wearable cyber-physical systems operating under resource constraints.

Therefore, the main contribution of this work is the integration of a lightweight CNN model into an embedded-oriented EMG processing framework for wearable CPS applications.

## 6. Analysis of Embedded Implementation Complexity

Embedded artificial intelligence is an important component of wearable cyber–physical systems, enabling low-latency operation, reduced reliance on external infrastructure, and improved data privacy. In EMG-based applications, local processing can minimize communication delays and support autonomous operation, which is particularly relevant for real-time wearable systems [7,12].

Low inference latency is essential in assistive and monitoring applications, where classification outputs may contribute to time-sensitive decisions. Previous studies have reported that excessive processing delays can negatively affect system responsiveness and user interaction quality [13,19]. Consequently, lightweight neural network architectures have become a key requirement for embedded wearable platforms.

The deployment of neural networks on embedded platforms is constrained by limited memory capacity, storage resources, processing power, and energy availability. Consequently, compact models are required to balance classification performance and computational efficiency in resource-constrained embedded environments [1,5]. Experimental validation in this study was performed on a BPI-M2-BERRY v1.0 platform. Typical hardware characteristics relevant to embedded AI deployment are summarized in Table 10.

The proposed 1D CNN architecture was designed to meet the memory and computational constraints commonly encountered in embedded wearable systems. After post-training int8 quantization, the model size was reduced from approximately 8 KB to 2 KB while preserving classification performance. With approximately 2000 trainable parameters, the model represents a compact solution suitable for resource-constrained environments [16].

From a computational perspective, the network requires approximately 3 × 10^4^ multiply–accumulate operations (MACs) per inference, indicating a relatively low computational burden compared with many deep learning approaches reported in the literature. Such characteristics support the feasibility of deployment on embedded-class hardware platforms.

In the experimental setup, EMG acquisition was performed using a MyoWare 2 Muscle Sensor Development Kit (KIT-27920; SparkFun Electronics, Niwot, CO, USA) connected to an Arduino MEGA (WIZI Electronics Co., Ltd., Shenzhen, China), while inference was executed on a BPI-M2-BERRY v1.0 platform (Shenzhen SinoVoIP Co., Ltd., Shenzhen, China). This configuration enabled validation of the complete signal-processing and classification pipeline under realistic operating conditions.

The main complexity indicators are summarized in Table 11.

Post-training quantization reduced the model size by approximately 75%, from 8 KB to 2 KB. Based on the computational requirements of the quantized model, the estimated inference latency on embedded hardware ranges between 5 and 12 ms. Although these values require experimental validation, they indicate suitability for near-real-time operation.

Energy efficiency is also important for wearable applications. Based on representative power consumption values reported for ARM Cortex-M devices, the energy required per inference is estimated to be on the order of several tens of microjoules, suggesting compatibility with low-power embedded operation.

Recent TinyML studies report EMG classification models with memory footprints ranging from several kilobytes to tens of kilobytes and computational requirements between 10^4^ and 10^6^ operations per inference [5,20]. In comparison, the proposed model occupies approximately 2 KB after quantization and requires about 3 × 10^4^ operations per inference, placing it among highly compact neural network models.

To further position the proposed approach, Table 12 presents a comparison with representative EMG classification models reported in the literature.

The comparison indicates that models achieving very high classification performance are often associated with substantially larger architectures and increased computational requirements. In contrast, the proposed model prioritizes computational efficiency and compactness while maintaining competitive classification performance.

Rather than maximizing accuracy alone, the proposed approach aims to achieve a balance between classification performance, memory footprint, and computational complexity, which are key requirements for future wearable cyber–physical systems operating under resource constraints.

## 7. Security, Resilience, and Ethical Considerations

The integration of artificial intelligence into EMG-based wearable cyber–physical systems introduces additional requirements related to reliability, security, and responsible system operation. Since these systems combine sensing, data processing, and automated decision-making, potential disturbances may affect both classification performance and overall system behavior.

Several factors can influence system reliability, including signal variability, electrode placement, motion artifacts, electromagnetic interference, and hardware-related constraints. In addition, wearable CPS deployments may be exposed to risks associated with software integrity, unauthorized modifications, or degraded prediction quality under non-ideal operating conditions.

To address these challenges, the proposed framework incorporates design principles aimed at improving robustness, including signal preprocessing, data validation, and monitoring of classification outputs. Additional mechanisms commonly adopted in embedded systems, such as secure boot, protected memory, integrity verification, and anomaly detection, may further enhance operational reliability.

Resilience is supported through the ability to detect uncertain or degraded operating conditions and to limit the impact of unstable predictions. Representative resilience mechanisms relevant to wearable CPS applications are summarized in Table 13.

In assistive applications, safety remains a primary design objective. For this reason, human supervision and user intervention should retain priority over automated decisions. Approaches such as bounded control actions, confidence monitoring, and timeout mechanisms can contribute to safer system operation in practical deployments.

From an ethical perspective, EMG signals constitute sensitive biometric information and require appropriate handling throughout the data lifecycle. In this study, data collection was performed based on informed consent, and all recorded data were anonymized before analysis. Furthermore, local processing reduces the need for external transmission of raw physiological data, contributing to improved privacy protection.

The proposed framework is also aligned with key responsible AI principles, including transparency, traceability, robustness, and human oversight. Representative dimensions relevant to wearable AI systems are summarized in Table 14.

Overall, security, resilience, and ethical considerations should be regarded as integral components of wearable cyber–physical system design. Their inclusion at the architectural level contributes to improving system reliability and supports the responsible deployment of AI-enabled EMG applications.

## 8. Discussion

### 8.1. Subject-Independent Validation Analysis

The proposed CNN achieved an accuracy of 0.9135 under the stratified train–validation split and a mean accuracy of 0.936 ± 0.011 under stratified 5-fold cross-validation, indicating stable classification performance across different partitions of the available dataset. While these protocols evaluate model consistency under controlled experimental conditions, the LOSO protocol provides a more demanding assessment by testing the model on previously unseen users. Under this subject-independent evaluation, lower performance was observed, highlighting the challenge of generalizing EMG-based movement recognition across different individuals.

It should also be noted that the ROC AUC value of approximately 0.96 was computed using the stratified train–validation evaluation protocol and therefore reflects segment-level classification performance. In contrast, the LOSO protocol evaluates subject-independent generalization and represents a substantially more challenging assessment scenario involving previously unseen participants. Consequently, the ROC AUC and LOSO results characterize different aspects of model performance and should not be interpreted as directly equivalent evaluation measures.

This behavior reflects the inherent inter-subject variability of EMG signals, which is influenced by factors such as muscle anatomy, electrode positioning, signal amplitude, and movement execution patterns [3,4,21]. Similar observations have been reported in previous EMG classification studies, where subject-independent evaluation is consistently more demanding than within-subject validation.

The confusion matrix analysis further showed that most classification errors occurred between walking and running activities. The similarity of their cyclic muscle activation patterns increases class overlap and makes discrimination more challenging, particularly in cross-subject evaluation scenarios.

From a practical perspective, the obtained results demonstrate that the proposed compact CNN can effectively classify lower-limb activities while maintaining low computational complexity. At the same time, the LOSO evaluation highlights the importance of subject-independent validation for wearable CPS applications intended for deployment in heterogeneous user populations.

Future work will focus on expanding the dataset, increasing participant diversity, and investigating techniques that improve cross-subject generalization while preserving the compactness and efficiency required for TinyML-oriented deployment.

### 8.2. Impact of Dataset Characteristics

The dataset used in this study was collected from 15 healthy participants performing squat, walking, and running activities. The relatively homogeneous participant group was selected to reduce physiological variability during the initial evaluation of the proposed EMG classification framework and to facilitate assessment of its feasibility under controlled conditions.

An additional characteristic of the dataset is the unequal distribution of validated samples across movement classes. The walking class contained fewer valid segments than the squat and running classes, primarily due to the signal-quality filtering procedure, which removed recordings affected by low amplitude, motion artifacts, or acquisition-related disturbances.

To minimize the impact of class imbalance, stratified data partitioning and stratified cross-validation were employed throughout the evaluation process. Furthermore, model performance was assessed using multiple complementary metrics, including precision, recall, F1-score, and ROC AUC. The consistent results obtained across these metrics suggest that the model maintained balanced classification performance despite the differences in class representation.

The relatively small number of participants may have contributed to the lower performance observed during subject-independent evaluation. Limited demographic and physiological diversity reduces the variability available during training and may affect the model’s ability to generalize to previously unseen users.

Therefore, the reported results should be considered a feasibility assessment of the proposed framework. Future studies should investigate larger and more heterogeneous datasets to further evaluate robustness and cross-subject generalization in real-world wearable applications.

### 8.3. Comparison with Existing EMG Classification Approaches

The rapid development of deep learning has significantly influenced EMG-based movement recognition, leading to the adoption of increasingly sophisticated neural network architectures. While traditional approaches rely on handcrafted features such as RMS, MAV, WL, or ZC, recent studies have shown that CNN-based models can automatically learn relevant signal representations and often achieve improved classification performance [9,12,13,14,15].

At the same time, higher performance is frequently accompanied by increased model complexity. Many state-of-the-art architectures incorporate a large number of parameters and require substantial computational resources, which may not represent a limitation for desktop or cloud-based applications but can become challenging in wearable and battery-powered systems.

To better position the proposed framework within the current research landscape, Table 12 presents a comparison with several representative EMG classification approaches reported in the literature. In addition to classification performance, the comparison includes implementation-related characteristics such as model size and computational complexity, which are particularly relevant for embedded and TinyML-oriented applications.

As shown in Table 12, the proposed model achieves competitive classification performance while maintaining a substantially smaller computational footprint than many previously reported deep learning solutions. Whereas some approaches rely on hundreds of thousands or even millions of parameters, the proposed architecture contains approximately 2000 trainable parameters and requires only 3 × 10^4^ MAC operations per inference.

These observations highlight an important trade-off between accuracy and implementation efficiency. Although larger models may achieve slightly higher recognition rates, compact architectures can offer advantages in terms of memory usage, computational cost, and deployment feasibility. From this perspective, the proposed framework demonstrates that effective EMG classification can be achieved with a lightweight architecture specifically designed for wearable cyber–physical systems operating under resource constraints.

### 8.4. Deployment Considerations and System Limitations

A key objective of this study was to assess whether a compact EMG classification model could support future deployment in wearable cyber–physical systems. The proposed framework combines microcontroller-based signal acquisition with AI-driven movement recognition, providing a practical foundation for lightweight EMG processing.

Experimental validation was performed using an Arduino MEGA-based acquisition platform and a BPI-M2-BERRY v1.0 inference platform. This configuration enabled end-to-end evaluation of signal acquisition, preprocessing, and classification under realistic conditions. However, the BPI-M2-BERRY v1.0 provides considerably greater computational resources than the low-power microcontrollers typically targeted by TinyML applications.

Therefore, the results should be interpreted as evidence of deployment feasibility rather than direct validation on MCU-class devices such as ESP32 or STM32. Similarly, latency and energy consumption were estimated from model complexity and hardware characteristics rather than measured directly.

Despite these limitations, the proposed framework demonstrates that compact neural networks can achieve a favorable balance between classification performance and computational efficiency, and the small memory footprint and low computational requirements indicate strong potential for wearable and battery-powered applications.

Future work will focus on validation on MCU-class platforms, including direct latency measurements, energy profiling, and long-term evaluation under real-world operating conditions.

### 8.5. Practical Implications for Wearable CPS

The results of this study demonstrate that reliable EMG-based movement classification can be achieved using a compact neural network architecture, without the need for large and computationally demanding deep learning models. This is particularly important for wearable cyber–physical systems, where memory, processing power, and energy resources are inherently limited.

An important finding is that effective movement recognition does not necessarily require increasingly complex architectures. Instead, a carefully designed lightweight model can provide a practical balance between classification performance and computational efficiency, making it suitable for embedded and battery-powered devices.

From an application perspective, the proposed framework has potential relevance in rehabilitation, assistive technologies, prosthetic control, activity monitoring, sports performance assessment, and human–machine interaction systems. In such scenarios, EMG-based movement recognition can support user intention estimation, activity tracking, and adaptive decision-making.

The study also highlights that deployment requirements should be considered alongside classification accuracy. For wearable systems expected to operate continuously and respond in real time, computational efficiency becomes just as important as recognition performance. Compact architectures can therefore offer significant practical advantages by reducing memory usage, computational load, and energy consumption.

Overall, the proposed framework demonstrates that lightweight AI solutions can support effective EMG-based movement recognition under realistic resource constraints, providing a practical foundation for future wearable CPS and TinyML-enabled applications.

## 9. Conclusions and Future Work

In this study, we presented a compact EMG-based movement classification framework for wearable cyber–physical systems, integrating signal acquisition, preprocessing, segmentation, and AI-driven recognition within a unified processing pipeline. The proposed approach was designed with embedded deployment requirements in mind, emphasizing computational efficiency and low resource consumption.

Our experimental results demonstrated that reliable movement recognition can be achieved using a lightweight neural network architecture. The proposed model achieved an accuracy of 0.9135 under stratified evaluation, a mean cross-validation accuracy of 0.936 ± 0.011, and a ROC AUC of approximately 0.96, while maintaining a compact memory footprint suitable for resource-constrained environments.

Our findings indicate that effective EMG classification can be achieved without relying on highly complex deep learning architectures. By combining satisfactory classification performance with low computational requirements, the proposed framework represents a practical solution for wearable applications where memory, energy efficiency, and real-time operation are important considerations.

Our study also highlighted the challenges associated with subject-independent EMG classification, emphasizing the importance of cross-user validation when assessing wearable AI systems intended for real-world deployment.

Future work will focus on expanding the dataset to include a larger and more diverse participant population, validating the framework on MCU-class platforms such as ESP32 and STM32, and performing direct latency and energy measurements on target hardware. Additional research directions include multimodal sensing integration and the incorporation of explainable AI techniques to improve transparency and support adoption in rehabilitation and assistive technologies.

## Figures and Tables

**Table 1:** Hardware limitations identified for the Arduino MEGA platform.

Parameter	Value
SRAM	8 KB
Flash memory	256 KB
Architecture	8-bit AVR
Clock frequency	16 MHz
TensorFlow Lite Micro compatibility	Limited

**Table 2:** CNN model architecture.

Layer	Configuration
Input	(32, 3)
Conv1D	8 filters, kernel size = 5
MaxPooling1D	pool size = 2
Conv1D	12 filters, kernel size = 3
MaxPooling1D	pool size = 2
Flatten	–
Dense	16 neurons, ReLU
Output	3 neurons, Softmax

**Table 3:** Performance metrics of the CNN model (mean ± standard deviation).

Metric	Value
Accuracy	0.9135
Precision	0.9097
Recall	0.9152
F1-score	0.9124
False positive rate	0.0600
Average inference time (desktop)	0.6000 ms
Estimated inference time (embedded)	5–15 ms

**Table 4:** Comparison with baseline machine learning models.

Method	Accuracy	Precision	Recall	F1-Score
SVM	0.88	0.87	0.89	0.88
Random Forest	0.90	0.89	0.90	0.89
k-NN	0.87	0.86	0.88	0.87
Proposed CNN	0.9135	0.9097	0.9152	0.9124

**Table 5:** Reported mean ± SD from the 5-fold validation.

Class	Precision	Recall	F1-Score
Squat	0.91	0.92	0.91
Walk	0.89	0.90	0.89
Running	0.92	0.93	0.92

**Table 6:** Stratified 5-fold cross-validation results.

Fold	Accuracy	Precision	Recall	F1-Score
1	0.93	0.92	0.90	0.91
2	0.94	0.93	0.91	0.92
3	0.92	0.91	0.89	0.90
4	0.95	0.92	0.91	0.91
5	0.94	0.92	0.90	0.91

**Table 7:** Leave-one-subject-out validation results.

Evaluation Protocol	Accuracy	Precision	Recall	F1-Score
Segment-level stratified split	0.9135	0.9097	0.9152	0.9124
Stratified 5-fold cross-validation	0.936 ± 0.011	0.920 ± 0.008	0.902 ± 0.009	0.910 ± 0.007
LOSO cross-validation	0.63 ± 0.04	0.59 ± 0.05	0.57 ± 0.04	0.54 ± 0.05

**Table 8:** Factors influencing model robustness.

Factor	Potential Impact	Mitigation Strategy
Sensor placement	Variations in signal amplitude	Signal normalization
Muscle fatigue	Temporal drift	Periodic recalibration
EMG noise	Misclassification	Filtering and CNN-based feature extraction
Rapid transitions	Prediction oscillations	Temporal smoothing

**Table 9:** Qualitative comparison of representative EMG classification approaches.

Study	Model Type	Application	Reported Performance	Model Complexity	Embedded Suitability
Rivière et al. [12]	Deep CNN	EMG biomedical analysis	High accuracy	High	Moderate
Chen et al. [13]	Multistream CNN	Gesture recognition	Improved performance	High	Limited
Prabhakar & Won [14]	ML classifiers	Finger movement classification	Competitive accuracy	Low–Moderate	High
Emimal et al. [15]	Multi-scale CNN	Prosthetic gesture recognition	Very high accuracy	High	Limited
Kim et al. [9]	Edge AI CNN	Embedded EMG recognition	Real-time capability	Moderate	High
Sahnoun et al. [16]	Lightweight CNN	Embedded gesture recognition	High accuracy with compression	Low	High
Sharma et al. [17]	Lightweight NN (TinyML)	Wearable IoT inference	Real-time inference	Low	High
Yamashita et al. [18]	CNN (TinyML)	Gait/time-series analysis	High accuracy	Low–Moderate	High
Proposed model	Compact CNN 1D	Wearable CPS EMG classification	Accuracy ≈ 0.91	Very low (~2000 params)	High

**Table 10:** Typical constraints in embedded implementation.

Parameter	Microcontroller Platform	Impact on AI Model
RAM	256 KB–520 KB	Limits model size and intermediate activations
Flash	1–4 MB	Limits number of parameters
CPU Frequency	80–240 MHz	Influences inference latency
Power Consumption	<500 mW	Requires energy-efficient design
Inference Time	<20 ms	Target for near real-time operation

**Table 11:** Complexity indicators of the optimized model.

Indicator	Float32 Model	Quantized Model (int8)
Parameters	2000	2000
Model size	8 KB	2 KB
MAC operations	3 × 10^4^	3 × 10^4^
Desktop latency (measured)	0.6 ms	0.6 ms
Embedded latency (estimated)	8–15 ms	5–12 ms

**Table 12:** Comparison between the proposed model and recent approaches in the literature.

Study	Task/Number of Classes	Accuracy	Parameters	Model Size	MAC/Inference	Notes
Proposed model	EMG classification (3 classes)	0.9135	2000	8 KB/2 KB (int8)	3 × 10^4^	Compact TinyML model
Taehun Kim et al. [9]	10 gestures	≈96%	≈171k	≈0.65 MB	≈1 × 10^6^	CNN + GRU, Edge AI
Rivière et al. [12]	3 EMG classes	≈89.5%	≈3M	≈11 MB	≈10^8^	CNN + LSTM
Prabhakar & Won [14]	15 movements	≈98.5%	N/A	N/A	N/A	Traditional ML
Emimal et al. [15]	NinaPro dataset	≈91–94%	N/A	N/A	N/A	Multi-scale CNN

**Table 13:** Representative resilience mechanisms for wearable CPS applications.

Mechanism	Role	Benefit
Fail-safe mode	Disable adaptive control	Prevent unintended actions
Hybrid control	AI + rule-based logic	Improved stability
Uncertainty monitoring	Detect unstable predictions	Reduced classification errors
Secure logging	Event traceability	Diagnostics and auditing

**Table 14:** Responsible AI dimensions.

Dimension	Implemented Measure
Robustness	Evaluation under signal variability
Traceability	Decision logging
Transparency	Model documentation
Human control	Manual override

## Data Availability

The data presented in this study are available from the corresponding author upon reasonable request.

## Abbreviations

The following abbreviations are used in this manuscript:

EMG	Electromyography
CNN	Convolutional Neural Network
1D CNN	One-Dimensional Convolutional Neural Network
CPS	Cyber–Physical System
AI	Artificial Intelligence
TinyML	Tiny Machine Learning
ADC	Analog-to-Digital Converter
ROC	Receiver Operating Characteristic
AUC	Area Under the Curve
FPR	False Positive Rate
TP	True Positive
FP	False Positive
TN	True Negative
FN	False Negative
MCU	Microcontroller Unit
TFLM	TensorFlow Lite Micro
UART	Universal Asynchronous Receiver–Transmitter
